# From childhood trauma to functional impairment in borderline personality disorder: the mediating role of emotion dysregulation and impulsivity

**DOI:** 10.3389/fpsyg.2026.1915829

**Published:** 2026-09-29

**Authors:** Jordi Morillas, Natalia Calvo, Nuria Gallardo, Marta Gil, Derek Clougher, Juan Jesús Crespín, Josep Antoni Ramos-Quiroga, Silvia Amoretti, Marc Ferrer

**Affiliations:** 1 Department of Mental Health, Vall d’Hebron Hospital Universitari, Barcelona, Spain; 2 Department of Psychiatry and Forensic Medicine, Universitat Autònoma de Barcelona, Spain; 3 Psychiatry, Mental Health and Addictions Group, Vall d’Hebron Research Institute (VHIR), Instituto De Investigación Sanitaria Acreditado Instituto De Investigación- Hospital Universitario Vall d’Hebron (IR-HUVH), Centro de Investigación Biomédica en Red de Salud Mental (CIBERSAM), The Carlos III Health Institute (ISCIII), Barcelona, Spain; 4 Group TLP Barcelona, Barcelona, Spain; 5 Bipolar and Depressive Disorders Unit, Hospital Clínic de Barcelona, Barcelona, Spain; 6 Departament de Medicina, Facultat de Medicina i Ciències de la Salut, Institut de Neurociències (UBNeuro), Universitat de Barcelona (UB), Barcelona, Spain; 7 Fundació Clínic-Institut d’Investigacions Biomèdiques August Pi i Sunyer (IDIBAPS), Barcelona, Spain; 8 Centro de Investigación Biomédica en Red de Salud Mental (CIBERSAM), The Carlos III Health Institute (ISCIII), Barcelona, Spain; 9 BIOARABA, Department of Psychiatry, Hospital Universitario de Álava, Centro de Investigación Biomédica en Red de Salud Mental (CIBERSAM), University of the Basque Country, Vitoria, Spain

**Keywords:** borderline personality disorder, childhood trauma, emotion dysregulation, mediation analysis, psychosocial functioning

## Abstract

**Background:**

Borderline Personality Disorder (BPD) is characterized by severe psychosocial dysfunction and high rates of adverse childhood experiences. Emotion dysregulation and impulsivity are core BPD features that may mediate the impact of childhood trauma on functioning.

**Methods:**

A total of 203 outpatients with BPD (mean age = 24.4 years; 84.7% women) completed the Childhood Trauma Questionnaire (CTQ), Difficulties in Emotion Regulation Scale (DERS), Barratt Impulsiveness Scale (BIS-11), and Functioning Assessment Short Test (FAST). Associations between childhood trauma (CTQ total and subscales) and psychosocial functioning were examined, testing the serial mediating role of the two mediators (PROCESS Model 6; percentile bootstrapping, 5,000 resamples; adjusted for gender and age).

**Results:**

CTQ total was associated with poorer functioning in the covariate-adjusted mediation model, with a significant direct effect. The total indirect effect through the mediators was significant, but no single pathway, including the serial CTQ → DERS → BIS-11 → FAST pathway, reached significance. Among trauma subtypes, emotional abuse and emotional neglect showed significant total and direct effects, and the serial pathway was significant for both neglect subtypes; sexual and physical abuse were not associated with functioning. When the five trauma subtypes were entered simultaneously, none showed a unique association.

**Conclusion:**

Childhood trauma is associated with poorer psychosocial functioning in BPD, partly through emotion dysregulation and impulsivity, although much remains unexplained. The absence of unique subtype effects points to the cumulative nature of childhood adversity (polyvictimisation). Being cross-sectional, these findings support trauma-informed therapeutic approaches only as hypotheses for future research.

## 1 Introduction

Borderline Personality Disorder (BPD) is a severe personality disorder characterized by a persistent pattern of instability in emotion regulation, interpersonal relationships, and self-image, as well as marked impulsivity (Diagnostic and Statistical Manual of Mental Disorders, Fifth Edition, Text Revision [DSM-5-TR]; American Psychiatric Association, 2022). Although the diagnostic criteria require symptoms to be present in early adulthood, their onset frequently occurs during adolescence, a particularly vulnerable period for the onset of psychopathology (Blakemore, 2019).

Numerous explanatory models of BPD have been proposed, among which Linehan’s biosocial model (Linehan, 1993, 2025) remains one of the most influential. This model posits that the development of BPD results from the interaction between a biological predisposition for emotion dysregulation and an invalidating social environment, characterized by inappropriate or dismissive responses to an individual’s emotional experiences, in turn contributing to chronic emotional dysregulation, a core feature of BPD.

According to this model, borderline pathology is understood to emerge from an ongoing transaction between a temperamentally based emotional vulnerability—characterized by heightened sensitivity, high reactivity, and a slow return to emotional baseline—and a pervasively invalidating developmental environment that dismisses, punishes, or trivializes the child’s emotional and private experiences (Crowell et al., 2009). When emotional expression is chronically invalidated, the child does not adequately learn to identify, label, understand, tolerate, and modulate affective states, and instead tends to oscillate between emotional inhibition and extreme emotional expression, giving rise to the persistent difficulties in emotion regulation that characterize BPD in adulthood (Crowell et al., 2009). Consistent with this account, childhood invalidation and harsh, invalidating parenting have been associated, both cross-sectionally and prospectively, with emotion dysregulation and borderline symptomatology (Keng and Soh, 2018; Hallquist et al., 2015).

Within the context of this model, a central concept of an invalidating social environment includes childhood maltreatment as an important risk factor for the development of BPD although it is not considered sufficient on its own to determine the disorder (Porter et al., 2020; Ibrahim et al., 2018). Among the different types of childhood maltreatment, studies conducted in both non-clinical (Kanj et al., 2023) and clinical samples (Krause-Utz et al., 2019) have predominantly identified emotional abuse as the subtype most strongly associated with BPD symptomatology. Kuo et al. (2015), who found that, after controlling other forms of abuse, emotional abuse uniquely explained the severity of borderline symptoms in a non-clinical population, conducted one of the earliest studies to explore this relationship. Subsequent research has replicated these findings in clinical samples (Rosenstein et al., 2018), suggesting that emotional abuse exerts both direct and indirect effects on BPD symptoms, whereas physical abuse shows a weaker association and the evidence regarding sexual abuse is mixed: although it is consistently associated with BPD at the level of exposure (Porter et al., 2020), its unique contribution to borderline symptomatology is frequently weak or non-significant once other maltreatment types are accounted for Kuo et al. (2015), Rosenstein et al. (2018). Nevertheless, not all individuals exposed to childhood maltreatment develop BPD (Temes et al., 2017).

This observation has driven further research aimed at identifying additional mechanisms that may explain the relationship between childhood trauma and borderline symptomatology. One of the most widely studied and empirically supported hypotheses proposes that difficulties in emotion regulation mediate this association (Thornton et al., 2025). A recent meta-analysis by Gruhn and Compas (2020) suggests that early childhood maltreatment disrupts the development of adaptive emotion regulation learning processes and promotes maladaptive strategies, such as emotional suppression, avoidance, and exaggerated emotional expression.

Consistent with this framework, recent studies have reported associations between emotional abuse and neglect and BPD symptoms in both clinical and non-clinical populations, mediated by difficulties in emotion regulation (Kanj et al., 2023; Schaich et al., 2021). However, these studies also converge in showing that the indirect effect through emotion dysregulation explains only a limited proportion of the direct effect of childhood maltreatment on borderline symptoms, highlighting the need to consider additional variables in this relationship to gain a clearer insight into the complex nature of this relationship.

Accordingly, other studies, such as that by Krause-Utz et al. (2019), drawing on Linehan (1993); biosocial model (2025), have observed in women with BPD that childhood maltreatment significantly impacts impulsivity through emotion regulation difficulties. Similarly, Schaich et al. (2021) suggested that behavioral difficulties in controlling impulses when distressed (that is, a behavioral expression of impulsivity rather than a specific subscale of the Difficulties in Emotion Regulation Scale [DERS]) may be the aspect of emotion dysregulation most closely linked to the association between emotional abuse and borderline symptoms, underscoring the role of impulsivity as a potential additional vulnerability interacting with emotion dysregulation.

Difficulties in regulating intense negative affect are, in turn, thought to heighten vulnerability to impulsive behavior. Within the framework of dialectical behavior therapy, when distress cannot be effectively modulated, individuals are more likely to operate from an “emotion mind” and to resort to maladaptive, impulsive actions–such as self-harm, substance use, or aggression–as short-term strategies to escape aversive emotional states (Linehan, 2025). This proposal is consistent with the emotional cascade model, in which rumination progressively amplifies negative affect until dysregulated, impulsive behaviors are enacted to interrupt the cascade (Selby et al., 2009), and with evidence that negative emotion dysregulation accounts for the association between BPD and impulsivity dimensions such as negative urgency (Fossati et al., 2014; Waite et al., 2024). Through these emotion-driven impulsive behaviors, early adverse experiences may ultimately contribute to impairment across interpersonal, social, and occupational domains.

Beyond the underlying mechanisms, there is broad agreement in the literature that BPD has a substantial impact on psychosocial functioning, with marked impairments in social, occupational, and academic domains that often persist across the lifespan, even when core borderline symptoms improve (Dhar et al., 2023). Moreover, an adolescent onset of BPD symptoms has been associated with poorer psychosocial functioning (Wall et al., 2021), increased mental health difficulties, greater risk of victimization, and worse therapeutic outcomes in adulthood (Temes et al., 2017; Wertz et al., 2020). Similarly, childhood maltreatment has been associated with poorer psychosocial functioning in later life both in clinical samples, with higher rates of mental disorders (Strathearn et al., 2020; Schneiderman et al., 2021), and in patients with BPD and non-suicidal self-injury (Ghinea et al., 2021). Together, these findings suggest that childhood trauma may be associated with long-term functional impairment in individuals with BPD. Although emotion dysregulation and impulsivity have primarily been examined in relation to borderline symptom severity, they may also contribute to psychosocial difficulties. Persistent difficulties in modulating emotional responses may compromise interpersonal and goal-directed functioning, whereas emotion-driven impulsive behaviors can destabilize relationships and interfere with educational and occupational continuity (Selby et al., 2009; Dhar et al., 2023; Waite et al., 2024). Emotion dysregulation and impulsivity may therefore represent complementary pathways through which childhood maltreatment is associated with real-world functional impairment in BPD. However, this potential trauma–mechanism–functioning pathway has received considerably less empirical attention than their role in borderline symptomatology.

Despite the clinical relevance of functional outcomes beyond symptom severity, there is a relative scarcity of validated instruments specifically designed to assess psychosocial functioning in individuals with BPD; the Functioning Assessment Short Test (FAST; Rosa et al., 2007), recently validated in adolescents and young adults with BPD (Calvo et al., 2025), is one of the few available instruments for this population. Moreover, findings regarding the mediating role of emotion dysregulation and impulsivity in the relationship between childhood maltreatment and functioning in BPD remain inconclusive, underlining an important gap in the extant literature.

Within this context, the present study aims to examine the differential associations between specific types of childhood trauma and psychosocial functioning in a sample of adolescent and adult outpatients diagnosed with BPD, and to explore whether these associations are mediated by emotion dysregulation and self-reported impulsivity.

## 2 Methods

### 2.1 Study design and setting

This was a cross-sectional, observational study conducted at the Mental Health Network of the Catalan Health Institute, specifically at the Psychiatry Department of Vall d’Hebron University Hospital (Barcelona, Spain).

### 2.2 Participants

Participants were recruited from the clinical population attending the Adolescent and Young Adult BPD Program at Vall d’Hebron Hospital. Eligibility criteria were: (i) age between 15 and 49 years; (ii) no intellectual disability or learning disorder; (iii) absence of current schizophrenia, bipolar I disorder, or active substance dependence disorder according to DSM-5-TR criteria; and (iv) no neurological or organic condition that could account for psychiatric symptoms. The study was approved by the Clinical Research Ethics Committee of Vall d’Hebron Hospital (PR (AG) 321/2015) and conducted in accordance with the Declaration of Helsinki and Good Clinical Practice guidelines. Written informed consent was obtained from all participants. For underage participants, assent was obtained together with written informed consent from their parents or legal guardians.

The absence of intellectual disability or learning disorder was established on the basis of clinical history and clinical judgement, in order to ensure that participants were able to understand the questionnaires; when borderline intellectual functioning was suspected, a cognitive assessment could be requested. Participants were informed in advance that childhood trauma would be assessed and agreed to take part. Assessments were embedded within ongoing clinical care, and emotional support or referral to specialized resources (e.g., a specialized childhood sexual abuse unit, community mental health services, or emergency services) was available if distress arose. No compensation was provided for participation.

### 2.3 Assessment

#### 2.3.1 Clinical and diagnostic assessment

The psychopathological evaluation was conducted in three consecutive sessions by a psychiatrist and a clinical psychologist. In the first session, conducted by the psychiatrist, written informed consent was obtained and a clinical interview was carried out to collect sociodemographic data and clinical history, and the Structured Clinical Interview for DSM-IV Axis I Disorders (SCID-I; First et al., 1996) was administered. In the second session, conducted by the clinical psychologist, the Structured Clinical Interview for DSM-IV Axis II Disorders (SCID-II; First et al., 1999) was administered together with the self-report questionnaires: the Childhood Trauma Questionnaire (CTQ), the DERS, and the Barratt Impulsiveness Scale (BIS-11). The third session, which was briefer and also conducted by the clinical psychologist, was used to complete the SCID-II, to administer the FAST, and to provide feedback and clarification of any doubts regarding the self-report questionnaires. The SCID-I and SCID-II were used to confirm the diagnosis of BPD and to assess current comorbidities according to DSM-IV and DSM-5-TR criteria. The self-report questionnaires (CTQ, DERS, and BIS-11) were administered in paper-and-pencil format; they were explained to participants beforehand and completed at home, and were reviewed with the clinician upon their return so that any doubts could be resolved and unanswered items completed, thereby minimizing missing and careless responses (Curran, 2015).

#### 2.3.2 Emotion regulation and impulsivity

Emotion regulation difficulties were assessed with the Spanish 28-item version of the DERS (Hervás and Jódar, 2008; original 36-item version by Gratz and Roemer, 2004). Items are rated on a 5-point Likert scale (1 = almost never; 5 = almost always) and summed into a total score (range 28–140), with higher scores indicating greater emotion dysregulation. The scale yields five subscales: lack of emotional awareness, lack of emotional clarity, non-acceptance of emotional responses, difficulties engaging in goal-directed behavior, and limited access to emotion-regulation strategies. Impulsivity was measured with the Spanish version of the BIS-11 (Patton et al., 1995; Spanish version, Oquendo et al., 2001), a 30-item self-report rated on a 4-point Likert scale (from rarely/never to almost always/always). Items are summed into a total score (range 30–120), with higher scores indicating greater impulsivity. The scale comprises three second-order subscales: attentional (cognitive), motor, and non-planning impulsiveness.

#### 2.3.3 Childhood trauma

Childhood adversity was evaluated with the 28-item short form of the CTQ (Bernstein et al., 1997); the validated Spanish version was used (Hernández et al., 2013). Items are rated on a 5-point Likert scale (1 = never true; 5 = very often true). Twenty-five items form five subscales (emotional abuse, physical abuse, sexual abuse, emotional neglect, and physical neglect), each scored from 5 to 25, yielding a total score from 25 to 125; higher scores indicate greater exposure to childhood maltreatment. Three additional items constitute a minimization/denial validity scale, which was recorded.

#### 2.3.4 Psychosocial functioning

Functional outcome was assessed using the FAST (Rosa et al., 2007; Calvo et al., 2025), a clinician-administered structured interview designed to evaluate psychosocial functioning during the previous 15 days. The FAST comprises 24 items rated on a 4-point Likert scale ranging from 0 (no difficulty) to 3 (severe difficulty), with higher scores indicating greater functional impairment.

#### 2.3.5 Reliability of measures

Internal consistency was estimated in R (RStudio) with the psych package. Because Cronbach’s α assumes tau-equivalence, McDonald’s ω was also computed from a single-factor model (Dunn et al., 2014). In the present sample both coefficients were closely comparable and ranged from good to excellent: CTQ total (α = 0.93, ω = 0.93) and its subscales (α = 0.67–0.94, ω = 0.71–0.94), DERS total (α = 0.93, ω = 0.94) and its subscales (α = 0.82–0.91, ω = 0.83–0.91), and FAST (α = 0.87, ω = 0.88). Item-level BIS-11 responses were not available in this dataset, so neither α nor ω could be estimated for this instrument in the present sample; the value shown in Table 1 is the internal consistency reported in its original validation (Cronbach’s α = 0.83; Patton et al., 1995). All coefficients are reported in Table 1.

**TABLE 1:** Internal consistency (Cronbach’s α and McDonald’s ω) of the study measures in the present sample.

Scale	Cronbach α	McDonald’s ω
CTQ total	0.93	0.93
Emotional abuse	0.88	0.88
Physical abuse	0.88	0.89
Sexual abuse	0.94	0.94
Emotional neglect	0.87	0.88
Physical neglect	0.67	0.71
DERS total	0.93	0.94
Awareness	0.82	0.83
Clarity	0.88	0.88
Non-acceptance	0.89	0.89
Goals	0.90	0.91
Strategies	0.91	0.91
BIS-11 total	0.83^a^	–
FAST total	0.87	0.88

Coefficients were computed in R (psych package) on the full sample (*n* = 203), except for the DERS total and the DERS clarity subscale, for which one participant had missing item-level data (*n* = 202). McDonald’s ω was estimated from a single-factor model and is reported alongside Cronbach’s α because α assumes tau-equivalence (Dunn et al., 2014). Item-level BIS-11 responses were unavailable, so the reported α was taken from the original validation study (Patton et al., 1995), and ω could not be estimated. The CTQ was scored following the 28-item Short Form (25 clinical items forming the five subscales plus a 3-item minimization/denial validity scale); the DERS is the 28-item Spanish version (Hervás and Jódar, 2008). Decimals are reported without a leading zero for coefficients that cannot exceed one. CTQ, Childhood Trauma Questionnaire; DERS, Difficulties in Emotion Regulation Scale; BIS-11, Barratt Impulsiveness Scale; FAST, Functioning Assessment Short Test; α, Cronbach’s alpha; ω, McDonald’s omega.

### 2.4 Statistical analyses

Data analyses were performed using IBM SPSS Statistics version 26. The distribution of continuous variables was tested with the Kolmogorov–Smirnov test (with Lilliefors correction), given the sample size (*n* > 50); because the statistical significance of the indirect effects was assessed using percentile bootstrapping, which does not assume normally distributed variables, the mediation inferences do not depend on this assumption (Mishra et al., 2019). Pearson correlation coefficients were first computed to explore bivariate associations between predictors, mediators, and outcome variables. Mediation analyses were then carried out to examine whether the association between childhood trauma, assessed using CTQ total and subscale scores, and psychosocial functioning, assessed using the FAST, was mediated by emotion dysregulation and impulsivity, measured using the DERS and BIS-11, respectively. Analyses were conducted with the PROCESS macro for SPSS (version 4.1; Hayes, 2022), specifically Model 6, which tests serial multiple mediation. For each model, we estimated the total effect of CTQ on FAST, the direct effect when accounting for the mediators, and the indirect effects via each individual mediator or serial pathway. The statistical significance of indirect effects was assessed using percentile bootstrapping with 5,000 resamples, with mediation considered significant when the 95% confidence interval (CI) did not include zero (Hayes and Rockwood, 2017).

In addition, to explore whether specific facets of the mediators accounted for the association, a parallel multiple-mediator model (PROCESS Model 4) was estimated with all five DERS subscales and all three BIS-11 subscales entered simultaneously as mediators of the CTQ total–FAST association. Model 4 was preferred over repeated Model 6 specifications because the eight facets are not ordered in a theoretically specified causal sequence; entering them in parallel estimates the unique indirect contribution of each facet while holding the remaining seven constant, whereas repeating Model 6 for each facet would have required an arbitrary causal ordering and multiplied the number of models tested. Each indirect effect therefore represents the contribution of that facet over and above the others; because inference for these indirect effects relies on bootstrap confidence intervals rather than *p*-values, no additional correction for multiple comparisons was required for this model. Finally, model fit for the serial models was examined using the Comparative Fit Index (CFI, values > 0.90 satisfactory), the Root Mean Square Error of Approximation (RMSEA, values < 0.05 satisfactory), and the Standardized Root Mean Square Residual (SRMR, values < 0.08 satisfactory) (Kline, 2015), derived with the lavaan package in R (Rosseel, 2012).

The serial mediation model is just-identified (saturated; zero degrees of freedom), so these indices are trivially optimal and are reported for completeness rather than as evidence of model fit.

All mediation models were adjusted for gender and age. Self-identified gender was the variable collected and recorded in four categories: women, men, non-binary, and transgender. Given the small cell sizes of non-binary and transgender individuals, these two categories were combined, yielding a three-level gender variable (women, men, non-binary/transgender). This variable was entered using dummy coding with women as the reference category, that is, two dummy variables (men versus women; non-binary/transgender versus women); a third indicator was not entered because it would be perfectly collinear with the intercept. Age was entered as a continuous covariate. Given the lack of association between educational attainment and functioning, it was not included as a covariate. For the trauma-subtype analyses, the total effects were corrected for multiple comparisons across the five CTQ subscales using the Benjamini–Hochberg false discovery rate (FDR).

Prior to the main analyses, we screened the data for multicollinearity, multivariate outliers, common method bias, and floor/ceiling effects. Multicollinearity among predictors and mediators was assessed with variance inflation factors (VIF) and tolerance (conventional thresholds of VIF > 5 or tolerance < 0.20 indicating concern). Multivariate outliers were examined with Mahalanobis distance against a χ^2^ criterion (*p* < 0.001). Common method bias was evaluated with Harman’s single-factor test; because item-level BIS-11 responses were unavailable, the test was conducted on the observed scale scores (the five CTQ subscales, the five DERS subscales, the BIS-11 total, and the FAST total) rather than on individual items, with a first unrotated factor accounting for less than 50% of the variance taken to indicate no substantial bias. Floor and ceiling effects were examined as the proportion of participants scoring at the minimum or maximum of each scale, and departures from normality were assessed with skewness and kurtosis. Four sensitivity analyses were performed to examine the robustness of the CTQ total model. First, the CTQ minimization/denial scale was entered as an additional predictor alongside CTQ total, to test whether under-reporting of maltreatment accounted for the findings. Second, participants scoring ≥ 1 on that scale were excluded. Third, the sample was restricted to participants older than 18 years, to test whether including adolescents influenced the estimates. Fourth, the analysis was restricted to women and men, using a single man-versus-woman dummy, to test whether the results depended on how the small non-binary/transgender group was handled. In addition, because maltreatment subtypes frequently co-occur, the five CTQ subscales were entered simultaneously as predictors of FAST, adjusting for gender and age, in order to examine their unique contributions.

## 3 Results

### 3.1 Demographic characteristics of the sample

The study included 203 participants diagnosed with BPD. The mean (M) age of the sample was 24.40 years (standard deviation [SD] = 8.23, range = 15–49). Regarding gender, 84.7% identified as women (*n* = 172), 13.8% as men (*n* = 28), 1.0% as non-binary (*n* = 2), and 0.5% as transgender (*n* = 1). Regarding educational attainment, more than half of the sample had completed compulsory secondary education (55.7%), while 17.7% had vocational training, 12.3% university studies, and a minority only primary education (8.9%). In terms of marital status, the vast majority were single (88.2%), with a small proportion married or living with a partner (7.9%) or separated/divorced (3.9%).

The mean CTQ total score was 54.52 (SD = 19.57). Subscale scores were as follows: emotional abuse (*M* = 14.90, SD = 6.22), physical abuse (*M* = 8.41, SD = 4.68), sexual abuse (*M* = 8.55, SD = 5.68), emotional neglect (*M* = 14.16, SD = 5.53), and physical neglect (*M* = 8.50, SD = 3.67). A detailed description of the sample, including clinical comorbidities (e.g., attention-deficit/hyperactivity disorder [ADHD] in 36.1% of the sample, and comorbid avoidant, depressive and paranoid personality traits), is provided in Table 2.

**TABLE 2:** Sample characteristics (demographic, clinical and questionnaire variables) of the study sample.

Variable	*n* (%) / M (SD)
Age, M (SD) [range]	24.4 (8.2) [15–49]
Gender (self-identified)	
Women	172 (84.7%)
Men	28 (13.8%)
Non-binary	2 (1.0%)
Transgender	1 (0.5%)
Educational attainment	
Compulsory secondary	113 (55.7%)
Vocational training	36 (17.7%)
University	25 (12.3%)
Primary (complete)	15 (7.4%)
Baccalaureate	11 (5.4%)
Primary (incomplete)	3 (1.5%)
Marital status	
Single	179 (88.2%)
Married/partner	16 (7.9%)
Separated/divorced	8 (3.9%)
Employment status	
Student	86 (42.4%)
Unemployed	59 (29.1%)
Employed	45 (22.2%)
Temp. sick leave	6 (3.0%)
Permanent disability	5 (2.5%)
Prison	2 (1.0%)
Comorbidity	
ADHD (any subtype)	73 (36.0%)
Comorbid personality disorders (SCID-II):	
Avoidant	82 (40.4%)
Depressive	67 (33.0%)
Paranoid	64 (31.5%)
Obsessive-compulsive	46 (22.7%)
Passive-aggressive	27 (13.3%)
Dependent	23 (11.3%)
Narcissistic	19 (9.4%)
Antisocial	14 (6.9%)
Histrionic	11 (5.4%)
Questionnaire scores	
CTQ total	54.52 (19.57)
DERS total	101.28 (21.43)
BIS-11 total	66.42 (17.10)
FAST total	27.82 (14.76)

Current Axis I comorbidity (SCID-I) was dominated by major depressive (*n* = 103; 50.7%) and anxiety (*n* = 53; 26.1%) disorders; substance-use disorders (*n* = 41; 20.2%), eating disorders (*n* = 19; 9.4%) and post-traumatic stress disorder (*n* = 14; 6.9%) were also represented. Categories are not mutually exclusive owing to comorbid presentations. ADHD, attention-deficit/hyperactivity disorder; SCID-I, Structured Clinical Interview for DSM-IV Axis I Disorders; SCID-II, Structured Clinical Interview for DSM-IV Axis II Disorders; M, mean; SD, standard deviation; CTQ, Childhood Trauma Questionnaire; DERS, Difficulties in Emotion Regulation Scale; BIS-11, Barratt Impulsiveness Scale; FAST, Functioning Assessment Short Test.

Preliminary analyses indicated no multicollinearity among predictors and mediators (all VIF < 1.4; tolerance > 0.72) and no influential multivariate outliers (no case exceeded the Mahalanobis χ^2^ criterion). Harman’s single-factor test, conducted on the scale scores (see Methods 2.4), indicated no substantial common method bias, with the first unrotated factor accounting for 21.9% of the variance. Floor and ceiling effects were negligible for the total scores entered in the primary models ( < 2% of participants at the minimum or maximum of the CTQ, DERS, BIS-11 and FAST). At the level of the CTQ subscales, however, marked floor effects were observed for sexual abuse (60.1% of participants scored at the scale minimum), physical abuse (43.3%) and physical neglect (25.1%), whereas floor effects were small for emotional abuse (8.9%) and emotional neglect (3.9%), and scores deviated only mildly from normality (all —skewness— and —kurtosis| < 1), supporting the use of bootstrapping.

### 3.2 Associations between childhood trauma and psychosocial functioning

At the bivariate level, emotional abuse, sexual abuse, emotional neglect, and physical neglect were each significantly correlated with psychosocial impairment as measured by the FAST, whereas physical abuse was not; the CTQ total score was also significantly correlated with the FAST. Poorer functioning was likewise associated with greater emotion dysregulation and impulsivity, as indicated by DERS and BIS-11 scores, respectively, and the CTQ total, DERS, and BIS-11 scores were significantly intercorrelated. Means, standard deviations, and all bivariate correlations are reported in Tables 2, 3.

**TABLE 3:** Pearson correlations among study variables.

Variable	CTQ total	Emotional abuse	Physical abuse	Sexual abuse	Emotional neglect	Physical neglect	DERS	BIS-11	FAST
1. CTQ total	–	–	–	–	–	–	–	–	–
2. Emotional abuse	0.85**								
3. Physical abuse	0.73**	0.57**							
4. Sexual abuse	0.64**	0.39**	0.34**						
5. Emotional neglect	0.78**	0.65**	0.40**	0.28**					
6. Physical neglect	0.77**	0.55**	0.55**	0.38**	0.61**				
7. DERS	0.15*	0.15*	−0.07	0.12	0.16*	0.20**			
8. BIS-11	0.19**	0.19**	−0.01	0.15*	0.20**	0.20**	0.51**		
9. FAST	0.27**	0.26**	0.11	0.17*	0.26**	0.21**	0.31**	0.36**	

Pearson correlations*;* *p < 0.05; **p < 0.01. Means and standard deviations are reported in Table 2 and are not duplicated here. CTQ, Childhood Trauma Questionnaire; DERS, Difficulties in Emotion Regulation Scale; BIS-11, Barratt Impulsiveness Scale; FAST, Functioning Assessment Short Test.

### 3.3 Serial mediation analyses

The serial mediation Model 6 tested whether childhood trauma was associated with psychosocial functioning, assessed using the FAST total score, through difficulties in emotion regulation, assessed using the DERS total score, and, in turn, impulsivity, assessed using the BIS-11 total score (see Figure 1). All models were adjusted for gender and age.

**FIGURE 1:**
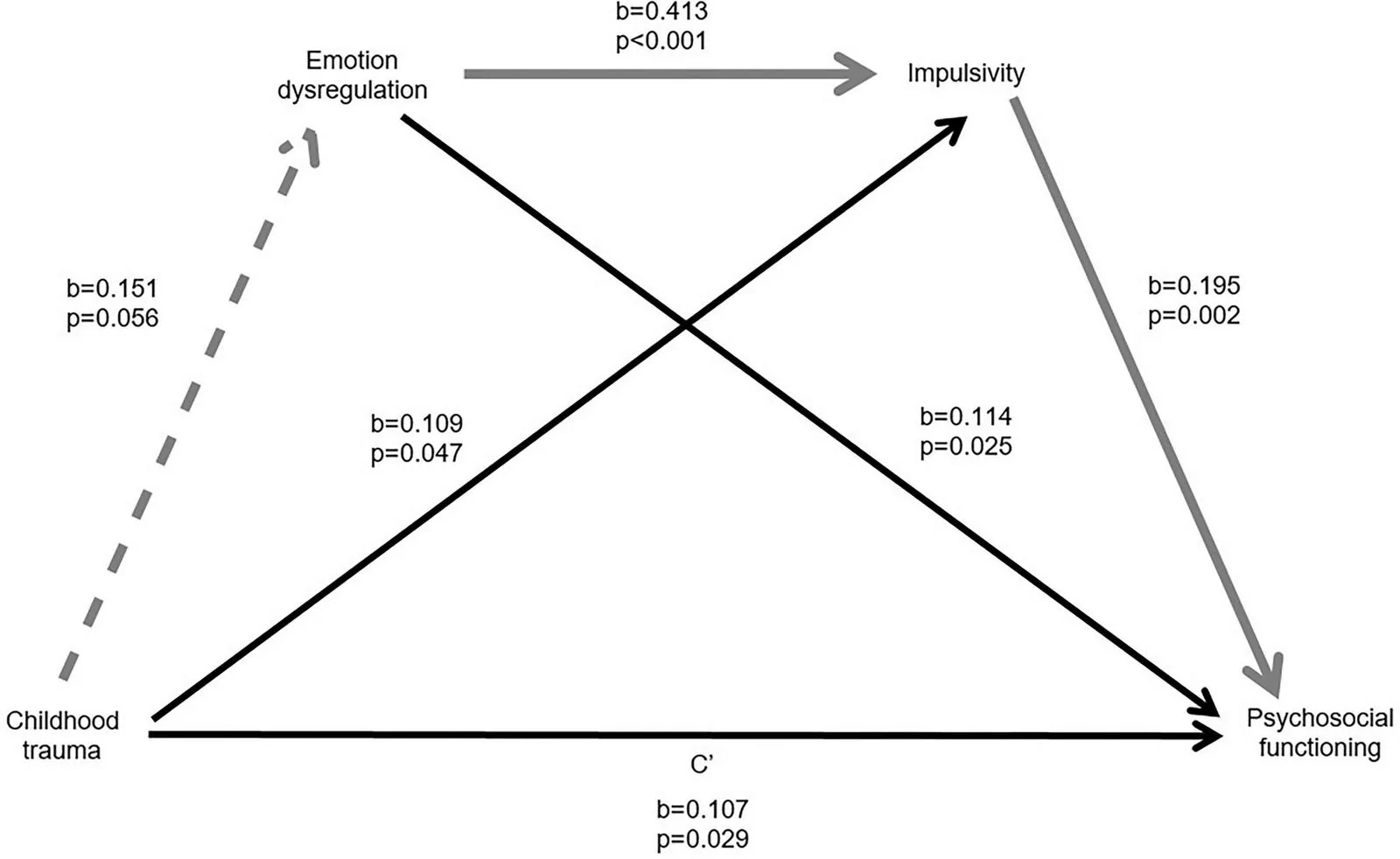
The serial mediating effect of emotion dysregulation and impulsivity in the relationship between childhood trauma and functionality. Unstandardized coefficients (b) with associated *p*-values; all paths were adjusted for gender (self-identified) and age. C′ is the direct effect of childhood trauma on psychosocial functioning. Solid arrows denote statistically significant paths (*p* < 0.05); dashed arrows denote non-significant paths. Constructs were assessed using total scores from the CTQ, DERS, BIS-11, and FAST, with higher FAST scores indicating greater functional impairment. The arrows show individual regression paths; indirect effects are reported in Table 4, where the serial indirect effect (CTQ → DERS → BIS-11 → FAST) was not statistically significant. CTQ, Childhood Trauma Questionnaire; DERS, Difficulties in Emotion Regulation Scale; BIS-11, Barratt Impulsiveness Scale; FAST, Functioning Assessment Short Test.

For CTQ total, the total effect on FAST was significant (*b* = 0.158, 95% CI [0.057, 0.258], *p* = 0.002). The direct effect was significant when both mediators were included (*b* = 0.107, 95% CI [0.011, 0.203], *p* = 0.029), and the total indirect effect was also significant (*b* = 0.051, 95% CI [0.009, 0.101]). However, none of the specific indirect pathways was individually significant: the pathway through DERS alone (*b* = 0.017, 95% CI [−0.002, 0.052]), the pathway through BIS-11 alone (*b* = 0.021, 95% CI [−0.001, 0.051]), and the serial pathway CTQ → DERS → BIS-11 → FAST (*b* = 0.012, 95% CI [−0.001, 0.030]) all included zero. Because the model is just-identified (saturated), the fit indices were trivially optimal (CFI = 1.00; RMSEA < 0.001; SRMR < 0.001) and therefore do not provide evidence of model fit. All effects are reported in Table 4.

**TABLE 4:** Serial mediation analysis (PROCESS Model 6): CTQ total predicting FAST.

Effect	b	*95% CI*	*p*
Total effect (c)	0.158	[0.057, 0.258]	0.002
Direct effect (c′)	0.107	[0.011, 0.203]	0.029
Total indirect effect	0.051	[0.009, 0.101]	–
CTQ → DERS → FAST	0.017	[−0.002, 0.052]	–
CTQ → BIS-11 → FAST	0.021	[−0.001, 0.051]	–
CTQ → DERS → BIS-11 → FAST	0.012	[−0.001, 0.030]	–

Unstandardized coefficients (b); 95% percentile bootstrap CIs (5,000 resamples), adjusted for gender and age. Total and direct effects are evaluated with *p*-values; indirect effects are evaluated with the bootstrap 95% CI and therefore have no associated *p*-value (–), being statistically significant when the CI does not include zero. Statistically significant effects are shown in bold. CI, confidence interval; CTQ, Childhood Trauma Questionnaire; DERS, Difficulties in Emotion Regulation Scale; BIS-11, Barratt Impulsiveness Scale; FAST, Functioning Assessment Short Test.

Trauma-subtype analyses revealed distinct patterns (see Table 5); total effects were corrected for multiple comparisons across the five subtypes (Benjamini–Hochberg FDR). Emotional abuse showed a significant total effect (*b* = 0.522, *p* = 0.001, FDR = 0.006) and a significant direct effect (*b* = 0.367, *p* = 0.016); its total indirect effect was significant (*b* = 0.154, 95% CI [0.030, 0.306]) and the indirect pathway through BIS-11 alone was significant (*b* = 0.068, 95% CI [0.002, 0.167]), whereas the serial pathway was non-significant (*b* = 0.036, 95% CI [−0.005, 0.090]). Emotional neglect showed significant total (*b* = 0.546, *p* = 0.003, FDR = 0.007), direct (*b* = 0.357, *p* = 0.039), and serial indirect (*b* = 0.051, 95% CI [0.001, 0.119]) effects. Physical neglect showed a significant total effect (*b* = 0.641, *p* = 0.019, FDR = 0.031) and a significant serial indirect effect (*b* = 0.105, 95% CI [0.026, 0.207]), whereas its direct effect was non-significant (*b* = 0.325, *p* = 0.212). Sexual abuse showed a non-significant total effect (*b* = 0.303, *p* = 0.095, FDR = 0.119); although its total indirect effect excluded zero (*b* = 0.141, 95% CI [0.011, 0.301]), no individual pathway was significant and the direct effect was non-significant. Physical abuse was not associated with functioning (total effect *b* = 0.183, *p* = 0.392).

**TABLE 5:** Serial mediation analyses (PROCESS Model 6): CTQ subtypes predicting FAST.

CTQ subtype	Total c (p; FDR)	Direct c′ (p)	Total indirect [95% CI]	Indirect via DERS [95% CI]	Indirect via BIS-11 [95% CI]	Serial indirect [95% CI]
Emotional abuse	0.522 (0.001; 0.006)	0.367 (0.016)	0.154 [0.030, 0.306]	0.051 [−0.008, 0.161]	0.068 [0.002, 0.167]	0.036 [−0.005, 0.090]
Physical abuse	0.183 (0.392; 0.392)	0.240 (0.226)	−0.057 [−0.261, 0.146]	−0.039 [−0.151, 0.056]	0.011 [−0.090, 0.127]	−0.029 [−0.109, 0.037]
Sexual abuse	0.303 (0.095; 0.119)	0.162 (0.342)	0.141 [0.011, 0.301]	0.041 [−0.023, 0.153]	0.071 [−0.000, 0.169]	0.030 [−0.018, 0.096]
Emotional neglect	0.546 (0.003; 0.007)	0.357 (0.039)	0.189 [0.045, 0.353]	0.070 [−0.006, 0.195]	0.068 [−0.016, 0.174]	0.051 [0.001, 0.119]
Physical neglect	0.641 (0.019; 0.031)	0.325 (0.212)	0.317 [0.098, 0.554]	0.135 [−0.003, 0.334]	0.077 [−0.048, 0.230]	0.105 [0.026, 0.207]

Unstandardized coefficients; 95% percentile bootstrap CIs (5,000 resamples), adjusted for gender and age. *P*, *p*-value for the total/direct effect; FDR-adjusted *p*-values were calculated using the Benjamini–Hochberg procedure across the five subtypes (total effects). Indirect effects (total and serial) are significant when the 95% CI does not include zero. Indirect via DERS = CTQ subtype → DERS → FAST; Indirect via BIS-11 = CTQ subtype → BIS-11 → FAST; Serial indirect = CTQ subtype → DERS → BIS-11 → FAST. Physical abuse was not associated with functioning; for sexual abuse the total effect was non-significant; for physical neglect the direct effect was non-significant. A model additionally including the CTQ minimization/denial scale as a predictor showed no association with functioning (total effect *b* = −1.43, *p* = 0.310). All estimates are reported to three decimal places; a confidence limit displayed as −0.000 is negative but smaller than 0.001 in absolute value, and the corresponding interval therefore includes zero. CTQ, Childhood Trauma Questionnaire; DERS, Difficulties in Emotion Regulation Scale; BIS-11, Barratt Impulsiveness Scale; FAST, Functioning Assessment Short Test; FDR, false discovery rate; CI, confidence interval.

Four sensitivity analyses were conducted. First, including the CTQ minimization/denial scale as an additional predictor showed no association with functioning (total effect *b* = −1.43, *p* = 0.310). Second, excluding participants flagged on that scale (score ≥ 1; *n* = 148 retained) yielded an essentially unchanged total effect (*b* = 0.137, *p* = 0.023) and direct effect (*b* = 0.106, *p* = 0.064). Third, restricting the sample to participants older than 18 years (*n* = 148) also left the coefficients essentially unchanged (total effect *b* = 0.142, *p* = 0.030; direct effect *b* = 0.102, *p* = 0.096). Fourth, restricting the analysis to women and men (*n* = 200) produced comparable estimates (total effect *b* = 0.154, *p* = 0.003; direct effect *b* = 0.106, *p* = 0.033). In all restricted samples the serial indirect effect was non-significant. In the two smaller subsamples (*n* = 148) the direct effect fell below the conventional significance threshold while its magnitude remained essentially unchanged, which is consistent with reduced statistical power rather than with a change in the underlying estimate. Finally, because maltreatment subtypes frequently co-occur, we additionally entered the five CTQ subscales simultaneously as predictors of FAST, adjusting for gender and age. Multicollinearity was acceptable (all VIF < 2.4), but no subtype retained a statistically significant unique association with functioning (emotional abuse *b* = 0.454, *p* = 0.058; physical abuse *b* = −0.400, *p* = 0.142; sexual abuse *b* = 0.128, *p* = 0.514; emotional neglect *b* = 0.218, *p* = 0.398; physical neglect *b* = 0.230, *p* = 0.538), indicating that the subtype-specific effects reported above reflect substantial shared variance among co-occurring forms of maltreatment.

Finally, a parallel multiple-mediator model (PROCESS Model 4) tested whether specific facets of the mediators accounted for the CTQ total–FAST association, with the five DERS subscales and the three BIS-11 subscales entered simultaneously as mediators (see Table 6). The total indirect effect across the eight facets was not significant (*b* = 0.043, 95% CI [−0.011, 0.102]), and no individual subscale emerged as a significant mediator (all bootstrap 95% CIs included zero).

**TABLE 6:** Parallel multiple-mediator model (PROCESS model 4): DERS and BIS-11 subscales as simultaneous mediators of CTQ total → FAST.

Mediator (facet)	a × b (indirect)	95% CI (bootstrap)	95% CI excludes 0?
DERS – Lack of awareness	0.006	[−0.013, 0.035]	No
DERS – Lack of clarity	0.007	[−0.008, 0.033]	No
DERS – Non-acceptance	−0.009	[−0.032, 0.006]	No
DERS – Goal-directed behavior	0.001	[−0.015, 0.020]	No
DERS – Limited strategies	0.008	[−0.017, 0.041]	No
BIS-11 – Attentional/cognitive	0.010	[−0.015, 0.042]	No
BIS-11 – Motor	0.002	[−0.024, 0.027]	No
BIS-11 – Non-planning	0.017	[−0.002, 0.047]	No

Unstandardized indirect effects (a × b) for each facet, entered simultaneously; 95% percentile bootstrap CIs (5,000 resamples), adjusted for gender and age. Total indirect effect across the eight facets = 0.043, 95% CI [−0.011, 0.102]. An indirect effect is considered statistically significant only when its 95% bootstrap CI excludes zero (see the “95% CI excludes 0?” column). No individual facet reached significance (all CIs included zero), so none uniquely mediated the CTQ–FAST association. Facet-specific estimates are rounded to three decimals; the total indirect effect is the model-based estimate and therefore does not equal the arithmetic sum of the rounded facet estimates. CTQ, Childhood Trauma Questionnaire; DERS, Difficulties in Emotion Regulation Scale; BIS-11, Barratt Impulsiveness Scale; FAS, Functioning Assessment Short Test; CI, confidence interval.

## 4 Discussion

The aim of the present study was to examine the role of specific types of childhood trauma on psychosocial functioning in a clinical outpatient sample of adolescents and adults diagnosed with BPD. In addition, we aimed to evaluate the mediating role of emotion dysregulation and impulsivity in this association.

Childhood trauma significantly correlated with poorer psychosocial functioning in this clinical sample, and this association was significant as a direct effect in the mediation model adjusted for gender, age and the proposed mediators. Emotion dysregulation and impulsivity together accounted for part of it: the total indirect effect was significant, although no single indirect pathway reached significance individually and a substantial proportion of the association remained unexplained by these mechanisms. At the bivariate level, all maltreatment subtypes except physical abuse correlated with poorer functioning, and emotion dysregulation and impulsivity were intercorrelated and each related to functioning. These results are consistent with previous studies that have examined the role of emotion dysregulation in relation to borderline symptomatology (Schaich et al., 2021; Kanj et al., 2023).

While emotion dysregulation and impulsivity may be relevant, existing evidence, and the present findings, suggest that they do not fully account for the association between early childhood trauma and psychosocial functioning. Accordingly, our results suggest that the emotion-dysregulation account of trauma-related impairment in BPD, that is, the framework whereby childhood adversity affects outcomes primarily through deficits in emotion regulation (Linehan, 2025; Schaich et al., 2021), may need to be broadened to incorporate additional mechanisms. When trauma subtypes were examined separately, different subtypes showed different patterns of direct and mediated associations, which is compatible with the possibility that the pathway from childhood trauma to functional impairment is not homogeneous. Because the design is cross-sectional and we did not model latent subgroups, these subtype-specific patterns should be read as preliminary, exploratory indications of heterogeneity rather than as evidence for distinct etiological subgroups (Andión et al., 2013; Laffite et al., 2025).

In the present study, emotional abuse and emotional neglect showed significant total and direct effects on psychosocial functioning. For emotional abuse, the indirect association involved impulsivity rather than the serial emotion-dysregulation → impulsivity pathway, whereas for emotional and physical neglect the serial pathway through emotion dysregulation and impulsivity was significant; for physical neglect the direct effect was non-significant in the mediation model, such that its association with functioning appeared to operate mainly through these variables. This pattern suggests that emotion dysregulation and impulsivity are relevant, but not uniform, correlates across trauma subtypes. When the five trauma subtypes were entered simultaneously none retained a unique association with functioning, which is consistent with polyvictimisation: maltreatment subtypes rarely occur in isolation (Porter et al., 2020; Laffite et al., 2025), and the subtype-specific estimates obtained from separate models should therefore not be read as evidence that any particular form of maltreatment is the specific driver of functional impairment. The broadly consistent association of emotional abuse and neglect with poorer functioning aligns with previous work (Kuo et al., 2015; Rosenstein et al., 2018) and might be related to poorer attachment quality and to hostile or maladaptive interpersonal models (Kanj et al., 2023; Warmingham et al., 2023; Zhang et al., 2024).

A distinct pattern emerged for sexual abuse. Although sexual abuse was correlated with functioning at the bivariate level, this association was not significant in the mediation analysis, where neither the direct effect nor any individual mediation pathway was significant. This may suggest that, in this sample, the association between sexual abuse and psychosocial functioning was weak, could be partly accounted for by covariates, and by shared variance with other maltreatment types. Any residual impact may operate through sequelae not captured by measures of emotion dysregulation and impulsivity, such as post-traumatic stress symptoms or dissociative processes; these were not assessed in the present study and remain hypothetical (Rosenstein et al., 2018; Kanj et al., 2023).

Physical abuse was not significantly associated with psychosocial functioning in our models, in line with previous studies reporting weak (Rosenstein et al., 2018) or non-significant (Kuo et al., 2015) associations with borderline symptom severity. It was also the only maltreatment subtype that was unrelated to either mediator at the bivariate level. These null findings should, however, be interpreted in the light of the distribution of the data: physical abuse and sexual abuse showed pronounced floor effects in this sample, with a large proportion of participants reporting no exposure, which restricts variance and reduces the statistical power available to detect associations for these subtypes. Beyond this, one possibility is that physical abuse is more strongly linked to externalizing outcomes, such as aggression and delinquency, than to the domains captured by the FAST (Strathearn et al., 2020). As noted above, once gender and age were taken into account in the mediation models, the direct effect of physical neglect on functioning was no longer significant, suggesting that its association with functioning may operate mainly through emotion dysregulation and impulsivity rather than directly.

Taken together, these findings suggest that emotion dysregulation alone does not account for the association between childhood trauma and functional impairment in BPD (Letkiewicz et al., 2021), and that considering impulsivity separately adds information. For the neglect subtypes, the association with functioning involved a serial pathway through emotion dysregulation and then impulsivity, consistent with attempts to manage intense emotional states within invalidating environments (Fossati et al., 2014; Liu, 2019; Waite et al., 2024). At the same time, the divergent subtype patterns raise the possibility that individuals with different trauma histories (e.g., predominant sexual abuse or complex trauma) may follow alternative pathways not primarily driven by emotion dysregulation and impulsivity. Testing this hypothesis would require person-centered approaches, such as latent profile or latent class analysis of maltreatment patterns (Laffite et al., 2025).

From a clinical perspective, these results are compatible with the possibility that patients with predominant histories of emotional abuse and neglect might benefit from emotion-regulation-focused interventions, and that those with significant physical neglect might benefit from programmes emphasizing behavioral activation, routine building and autonomy training. Individuals with histories of sexual abuse might in turn benefit from trauma-focused components in addition to emotion-regulation strategies. However, because the study did not assess treatment response, these considerations should be regarded as hypotheses for future intervention research rather than as clinical recommendations directly derived from the present findings.

The present findings should be considered in the light of the study’s strengths and limitations. Strengths include a well-characterized clinical sample of adolescents and adults with BPD, the use of a clinician-administered measure of psychosocial functioning, correction for multiple comparisons across trauma subtypes, and several sensitivity analyses whose results were consistent with the main findings. Nevertheless, several limitations should be acknowledged.

First, the cross-sectional design precludes establishing the temporal ordering assumed by the mediation models and, consequently, drawing causal conclusions about the relationships among childhood trauma, emotion dysregulation, impulsivity, and psychosocial functioning. In addition, the absence of a healthy or clinical control group prevents determining whether the observed associations are specific to BPD or reflect broader transdiagnostic relationships between childhood adversity, emotion regulation, impulsivity, and functioning. Longitudinal studies including appropriate comparison groups are needed to establish temporal precedence and examine the diagnostic specificity of these findings.

Second, psychiatric comorbidities, which are highly prevalent in individuals with BPD and may independently contribute to psychosocial impairment (Eaton et al., 2011), were not included as covariates. Consequently, the potential influence of comorbidity on the observed associations could not be disentangled. This may be particularly relevant for ADHD, which was present in over one-third of the sample and is itself characterized by impulsivity and executive-function difficulties that may contribute to both BIS-11 scores and psychosocial functioning. Future studies with sufficiently large samples should assess the potential contribution of major comorbidities through covariate adjustment or appropriately powered stratified analyses.

Third, most participants were women, reflecting the composition of many treatment-seeking BPD samples, and the sample covered a broad age range of 15–49 years. Although all models were adjusted for gender and age, only 28 participants were men, precluding adequately powered analyses of potential gender-specific patterns. The broad age range may also have introduced developmental heterogeneity, as the clinical expression of BPD features, the consequences of childhood maltreatment, and the demands involved in psychosocial functioning may differ substantially between adolescence, emerging adulthood, and later adulthood (Li et al., 2025).

Fourth, childhood maltreatment, emotion dysregulation, and impulsivity were assessed exclusively through self-report. Although the CTQ, DERS, and BIS-11 are well-established instruments with strong psychometric properties, self-report measures remain vulnerable to social desirability, limited insight, and current affective state (Baker and Ben-Ami, 2011). The retrospective assessment of childhood maltreatment may also be affected by recall bias in individuals with BPD (Brewin, 2011). In addition, the CTQ physical neglect subscale showed the lowest internal consistency among the study measures, and findings involving this subtype should therefore be interpreted with caution. Future research would benefit from multimethod assessments combining self-reports with behavioral emotion-regulation or stress-reactivity paradigms, behavioral measures of impulsivity such as Go/No-Go, Stop-Signal, or delay-discounting tasks, and investigator-based interviews of childhood adversity, such as the Childhood Experience of Care and Abuse interview (Bifulco et al., 1994).

In conclusion, in this cross-sectional sample of adolescents and adults with BPD, childhood trauma was associated with poorer psychosocial functioning. Emotion dysregulation and impulsivity accounted for part of this association, most consistently for the neglect subtypes, but a substantial proportion remained unexplained and several indirect pathways were not statistically significant. Moreover, when all maltreatment subtypes were considered together none retained a unique association with functioning, consistent with the cumulative nature of childhood adversity. Because the design is cross-sectional, these findings are associational and do not support causal or clinical conclusions. They suggest, as hypotheses for future longitudinal research, that explanatory models of trauma-related functional impairment in BPD may need to consider mechanisms beyond emotion dysregulation and impulsivity.

## Data Availability

The data analyzed in this study is subject to the following licenses/restrictions: The data that support the findings of this study are available upon reasonable request to the corresponding author. Requests to access these datasets should be directed to Natalia Calvo, natalia.calvo@vallhebron.cat.
